# Editorial: Genomic insights and molecular genetics of plant-nematode interactions

**DOI:** 10.3389/fpls.2026.1978293

**Published:** 2026-09-09

**Authors:** Margarida Espada, Marie Manasová, Cláudia S.L. Vicente

**Affiliations:** 1 MED Mediterranean Institute for Agriculture, Environment and Development & CHANGE Global Change and Sustainability Institute, Institute for Advanced Studies and Research, Universidade de Évora, Évora, Portugal; 2 Department of Plant Protection, Faculty of Agrobiology, Food and Natural Resources, Czech University of Life Sciences, Prague, Czechia

**Keywords:** effector biology, genomic and transcriptomic analysis, nematode-host interactions, plant defense mechanisms, sustainable nematode management

Plant-parasitic nematodes (PPNs) affect agricultural and forest ecosystems worldwide, causing substantial economic losses and posing a challenge to sustainable crop and forestry production (Khan, 2025). The successful parasitism of PPNs is the outcome of a molecular dialogue with their hosts - as they exploit molecules and modulate host cellular processes for their own benefit. In response, plant hosts trigger defence molecules and nematode-derived signaling to restrain parasitism processes (Espada et al., 2025; Bell et al., 2026). Comparative genomics and integrative studies using multiomics approaches such as single-cell/spatial transcriptomics, proteomics, metabolomics, genome-wide association studies, will continue to reveal parasitism mechanisms and molecular interactions between nematodes and their hosts and support the development of sustainable strategies for efficient control of plant pathogens. These technologies have uncovered key mechanisms underlying nematode parasitism, host defense responses, genetic and genomic adaptations, while providing valuable resources for identifying resistance genes, molecular markers, and targets for innovative control strategies. The four contributions presented in this Research Topic showed recent advances in plant-nematode interactions, unveiling on nematode biology and genomics, as well as the molecular, physiological, and metabolic responses of plant hosts to infection.

Using a genome-wide association analysis, Inácio et al. explored the genetic architecture underlying the response of *Pinus pinaster* to the *Bursaphelenchus xylophilus* infection. Evaluating multiple half-sib pine families and thousands of single nucleotide polymorphisms (SNPs), the authors identified genomic regions associated with disease progression and susceptibility. Candidate *loci* were linked to genes involved in chloroplast function, immune signaling, and stress responses, while the analyses also revealed the importance of non-additive genetic effects, including epistasis and overdominance, in determining host response. These findings provide new molecular insights that may support marker-assisted selection and accelerate breeding programs for pine wilt disease resistance, representing an important contribution to forestry tree management.

The establishment of nematode infection relies on the secretion of effector proteins produced in the parasitism-specialized pharyngeal gland cells. Li et al. investigated the sulfate permease (SULP) gene family in the pinewood nematode *B. xylophilus*, the causal agent of pine wilt disease. Through comprehensive genome-wide characterization, the study identified ten members of the SULP family and analyzed their phylogenetic relationships, structural organization, chromosomal distribution, and expression patterns throughout the nematode life cycle. Their findings revealed differential expression of several *Bx-sulp* genes during developmental stages associated with stress adaptation, particularly in *dauer* stage, suggesting an important role in environmental adaptation and nematode fitness. This work identified new potential molecular targets for strategies to control *B. xylophilus*.

Host responses to nematode infection were also characterized using different omics approaches. Mengxin et al. performed a comparative transcriptomic analysis of resistant and susceptible tomato cultivars infected with the root-knot nematode *Meloidogyne incognita*. By analyzing gene expression profiles across multiple infection stages, the authors demonstrated that early infection stages are critical for distinguishing resistant and susceptible responses. Their results highlighted the activation of plant-pathogen interaction pathways in the resistant cultivar, whereas hormone signaling pathways predominated in the susceptible genotype. The study identified the phenylpropanoid biosynthesis pathway as a major component of tomato resistance, highlighting its potential role in reinforcing plant defenses and selected candidate genes for the development of improved nematode-resistant cultivars.

Zheng et al. adopted an integrated multiomics strategy to investigate the response of the medicinal plant *Trichosanthes kirilowii* to *M. incognita* infection. By combining physiological measurements, hormone profiling, transcriptomics, and metabolomics, the authors generated a comprehensive overview of the molecular and metabolic reprogramming triggered during nematode infection. Their integrative analyses identified zeatin biosynthesis as a main regulatory pathway coordinating hormonal responses and defense-related metabolic adjustments. This study showed that by combining multiple omics tools we can identify key regulatory pathways that may remain undetected using individual approaches.

As sequencing technologies, computational biology, and genome editing continue to evolve, our ability to understand the complexity of plant-nematode interactions will further accelerate, opening new opportunities for protecting agricultural and forest ecosystems against one of the most persistent biological threats. Moreover, understanding the biology of nematode effectors will facilitate the development of innovative management and control strategies that reduce reliance on synthetic nematicides while improving global food security.

## References

[B1] Bell C. A. Derevnina L. Eves-van den Akker S. (2026). Cross-kingdom communication between plants and parasitic nematodes. New Phytol. 251, 89–93. doi: 10.1111/nph.71283 42132224

[B2] Espada M. Vicente C. S. L. Vieira P. (2025). Molecular insights into migratory plant-parasitic nematodes. Annu. Rev. Phytopathol. 63, 403–430. doi: 10.1146/annurev-phyto-112624-103056 40903421

[B3] Khan M. R. (2025). “ Plant-parasitic nematodes impacting global food security”, in Nematode Disease Complexes in Agricultural Crops ( Wallingford, United Kingdom: CABI digital library), 1–32. doi: 10.1079/9781800625228.0001

